# Correlation between the density of defect states (DDS) and cross-linking of corner/edge sharing GeSe_4_ tetrahedral structural units

**DOI:** 10.1016/j.heliyon.2023.e21424

**Published:** 2023-10-30

**Authors:** Shiv Kumar Pal, A. Dahshan, Neeraj Mehta

**Affiliations:** a Department of Physics, Institute of Science, Banaras Hindu University, Varanasi, 221005, India; b Department of Physics, Faculty of Science, King Khalid University, P.O. Box 9004, Abha, Saudi Arabia

**Keywords:** Amorphous materials, Defects, Density of defect states, Electrical properties, A.C. conductivity

## Abstract

We have estimated the DDS in the STSG [Se_78-x_Te_20_Sn_2_Ge_x_ (x = 0, 2, 4, 6)] system by using the Correlated Barrier Hopping (CBH) model by performing A.C. conduction measurements in the frequency range (1 kHz–10 kHz) and temperature underneath the glass transition temperature (303–333) K. The detailed analysis reveals that bi-polaron hopping is a leading conduction mechanism over single-polaron hopping. Further, there is a noticeable reduction in DDS with increasing concentration of Ge beyond the composition x = 2. A close inspection indicates that cross-linking of Se with Ge has an important role in controlling the DDS in terms of the corner/edge sharing configurations in the structural unit of GeSe_4_ tetrahedral.

## Graphical abstract

**Figure:**
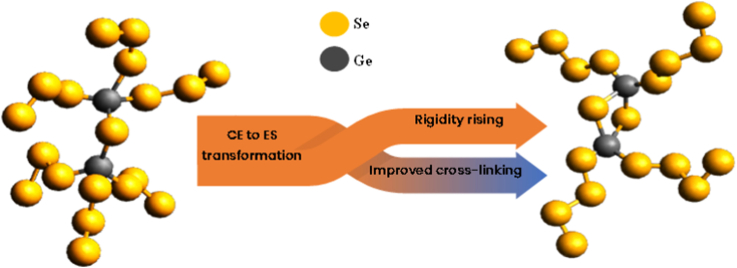

## 1 Introduction

The explanation of alteration in different physical properties of materials by finding their correlation with structure is a more convincing way in different scientific fields [1,2]. The study of any relationship between the structure and property helps in choosing a certain glass for an anticipated property [3,4]. However, it is not an easy task and it needs a comprehensive analysis of different physical properties to ensure their variation with a specific structural modification for a given material or a system of materials. The glasses Ge_x_Ch_100–x_ of Ge with chalcogen elements Ch (Ch = Se, Te, S) are the ideal candidates that demonstrate the structure-property inter-relations [5]. Keeping in mind this point, we have studied various physical properties of the present samples of the STSG system and reported the corresponding results in Refs. [[6], [7], [8], [9]]. The results of different optical, thermal, electrical, and mechanical properties of this system are reached at a common conclusion that the structural rigidity of the aforesaid glasses is increased drastically when we increase Ge content beyond x = 2. The reason behind such rigidity percolation appears as a rise in edge-sharing structural units of GeSe_4_ tetrahedral structural units over the corner-sharing ones.

When it comes to mid-infrared (IR) applications, chalcogenide glasses (ChGs) belong to an unrivaled class of glassy alloys. Despite the fact that compared to oxide glasses, this class of glasses is still in its infancy, ChGs have garnered notable attention due to their distinctive physical/chemical characteristics and intriguing applications [10]. ChGs have attracted the interest of numerous research teams from all over the world in recent years as a result of their exciting, innovative, and enormously important sensing applications in diverse fields of science and industry.

It has been discovered that using fiber evanescent wave spectroscopy techniques and optical fibers, solid-state thin films, and lenses of ChGs can be used in various types of applications [[11], [12], [13]]. ChGs-based sensors contain several unique instances of sensing applications that could be used in biosensing and food security [[11], [12], [13]].

The density of defect states plays a crucial role in non-crystalline solids, also known as amorphous or disordered materials [14]. Defects in amorphous materials can arise due to structural disorder, impurities, vacancies, or other irregularities [15]. It's worth noting that the exact nature of defect states in non-crystalline solids is still a topic of active research, and the understanding of their behavior continues to evolve. Experimental investigations, theoretical modeling, and computational simulations are employed to gain insights into the role of defect states and their impact on the properties of these materials.

In disordered materials, the nature of the conduction mechanism that takes place is affected by defect states. It was reported that the incorporation of Ge in parent glassy systems plays a crucial role i.e., it remarkably affects their optical, electrical as well as thermal properties. The variation in different parameters is mainly due to changing of charge defect states by the addition of a chemical modifier. In amorphous semiconductors, to estimate the value of the DDS, the analysis of A.C. conductivity *σ*_*ac*_ becomes more essential and it is utilized as a powerful tool to find information about the DDS. A painstaking survey shows that sufficient data related to the *σ*_*ac*_ is available in the literature for Ge-containing glasses to explain its behavior with the rise in frequency/temperature [[16], [17], [18], [19], [20], [21], [22]]. In this present paper, we have calculated the DDS of the STSG system and found that its composition dependence is directly correlated to cross-linking of corner/edge-sharing GeSe_4_ tetrahedral structural units.

## 2 Experimental section

The glassy alloys of present the STSG system were prepared by using melt-quench of each sample enclosed in a quartz tube sealed at 10^−6^ Torr. Before quenching in chilled water, the appropriate heat treatment of each tube was done in a furnace. The cylindrical-shaped pellets of the samples were used for electrical measurement in a metallic sample holder under a vacuum of 10^−3^ Torr. We have used a digital LCR meter (Wayne Kerr Electronics, UK; Model: 43100) attached in the dielectric measurements assembly in capacitance-dissipation factor (i.e., C_p_ – D) mode for the purpose of dielectric measurements of this synthesized ChGs. In this case, the cylindrically shaped pellets behave like a parallel arrangement of capacitor C and resistance R. Further, a sinusoidal voltage possessing frequency f (where f = ω/2π; ω is angular frequency) across the sample has been applied. We have measured the parallel capacitances (C and C_0_) and dissipation factor (D) at different temperatures and frequencies and subsequently, the values of dielectric constant (ε′) and loss (ε") can be calculated using these formulae [23]:

(1) $${ԑ}^{\prime }=\frac{C}{C_{0}}$$

and

(2) $${ԑ}^{\prime \prime }=D{ԑ}^{\prime }$$

## 3 Theoretical basis

The expression of maximum barrier height (W_m_) can be written as:

(3) $$W_{m}=\frac{6k_{B}T}{\left(1-s\right)}-k_{B}Tln\left(\tau_{0}\omega \right)$$

Here, *k*_*B*_ is Boltzmann constant, ‘s’ is the frequency exponent which is calculated by the slope of the plot between ln*σ*_*ac*_ versus ln*ω*. Also, ω is the angular frequency, *τ*_*0*_ is the characteristic relaxation time of the order of atomic vibrational period (∼10^−12^ s) and T is the temperature [[23], [24], [25]]. From equation (3), it is clear that the maximum value of ‘s’ will be one at temperature T = 0 K and it decreases with increasing temperature.

The minimum hopping distance can be expressed by the following relation-

(4) $$R_{\min}=\frac{ne^{2}}{\pi ԑ{ԑ}_{0}W_{m}}$$

in Eq. (4), *n* = 1 for single and *n* = 2 for bi-polarons. The values of Coulombic potential barrier height W can be calculated as-

$$W=W_{m}-\;\frac{ne^{2}}{\pi ԑ{ԑ}_{0}R}$$

Where *R* is the distance between neighboring sites, ԑ is the bulk dielectric constant, ԑ_0_ is the permittivity of free space and e is the electronic charge.

The expressions of AC conductivity for the bi-polarons hopping originating from D^+^ - D^−^ pairs taking as non-random distribution can be written according to the CBH model as-

(6) $$\sigma_{ac}\left(\omega \right)=\frac{2\pi^{3}}{6}NN_{p}ԑ{ԑ}_{0}\omega R_{\omega }^{6}\;\exp\left(\frac{e^{2}}{4\pi ԑ{ԑ}_{0}R_{\omega }k_{B}T_{g}}\right)$$

The density of the localized state at which the carriers exist is represented by *N*, and to which the carriers hop is given by *N*_*p*_, *T*_*g*_ is glass transition temperature and *R*_*ω*_ is the hopping distance for the case ωτ_0_ = 1, i.e., when ln (*ωτ*_0_) tends to zero.

The expression for *R*_*ω*_ is given below:

(7) $$R_{\omega }=\frac{ne^{2}}{\pi ԑ{ԑ}_{0}W_{m}}{\left(1+\frac{k_{B}T}{W_{m}}\ln\left(\omega \tau_{0}\right)\right)}^{-1}$$

In the Correlated Barrier Hopping (CBH) model, the hopping distance refers to the spatial separation between two neighboring sites or positions in a material where charge carriers can hop. The hopping distance can vary depending on the specific material and the properties of the disorder present. In some cases, it can be equal to the order of lattice constant or atomic spacing, while in other cases, it can be larger, spanning several unit cells, or even longer distances. The magnitude of the hopping distance is influenced by factors such as the strength of the disorder, the density of available hopping sites, and the energy landscape of the material.

The expressions of AC conductivity for the single polaron hopping originating from randomly distributed defect centers can be written as [[23], [24], [25]]:

(8) $$\sigma_{ac}\left(\omega \right)=\frac{\pi^{3}}{6}NN_{p}ԑ{ԑ}_{0}\omega R_{\omega }^{6}$$

## 4 Results and discussion

**Fig. 1:**
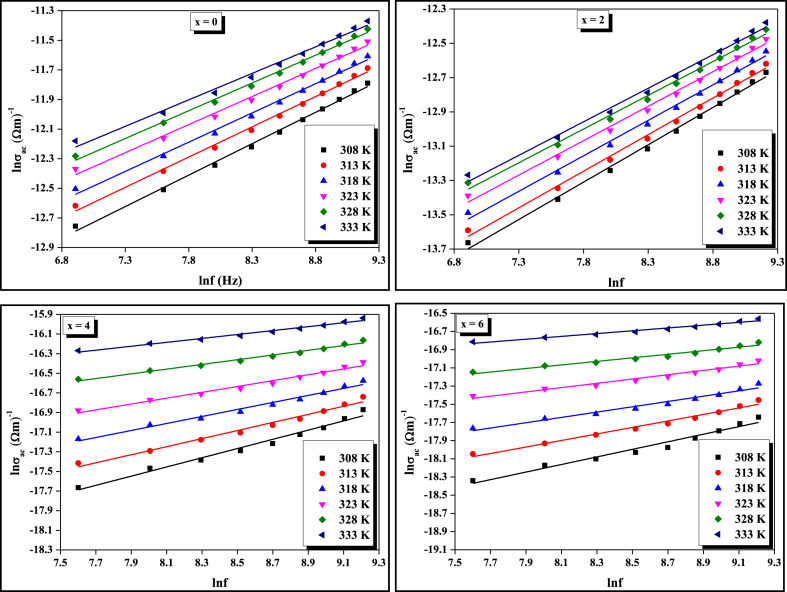
Frequency dependence of A. C. conductivity at different temperatures for all four compositions.

**Fig. 2:**
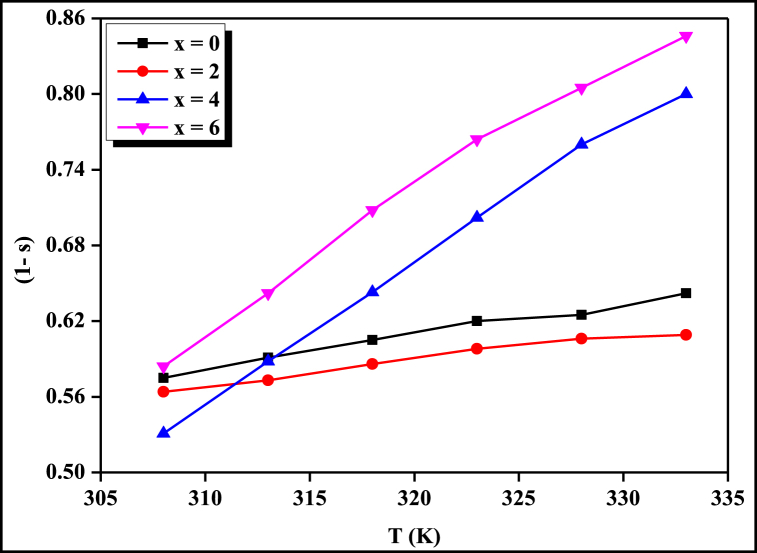
Temperature dependence of frequency exponent (s) for all four compositions.

The behavior of the dielectric parameters (i.e., dielectric constant and loss) with varying frequency and temperature was already reported in Ref. [6] for the present STSG system. However, the knowledge of A.C. conduction is essential for the estimation of DDS. In this endeavor, therefore, we have focused our attention on the determination of *σ*_*ac*_ and its variation with varying temperature/frequency. The frequency variation of *σ*_*ac*_ obeys a universal power law of the form *σ*_*ac*_ *(ω, T)* = *A(T). ω*^*s(ω, T)*^. The variation of *σ*_*ac*_ against frequency *f* at different temperatures for four compositions is represented in Fig. 1. From the linear plots of ln*σ*_*ac*_ against *lnω*, we have calculated the values of frequency exponent (s) and constant (A) using slope and intercept respectively at different temperatures for four compositions which are given in Table 1. The value and nature of the frequency exponent (*s*) with temperature indicate that the A.C. conduction mechanism of the present STSG glassy system obeys the CBH model [26,27] because the value of frequency exponent (s) at different temperatures is less than one and it decreases with increasing temperature which is an essential condition for holding CBH model. The variation of (1 – s) against *T* for four compositions is depicted in Fig. 2.

**Table 1:** Values of frequency exponent s (slope) and constant (A) in the plot of *lnσ*_*ac*_ versus *lnf*.

T (K)	x = 0		x = 2		x = 4		x = 6	
	s	A	s	A	s	A	s	A
308	0.425	6.8 × 10^−8^	0.436	2.5 × 10^−8^	0.469	2.3 × 10^−10^	0.416	2.1 × 10^−10^
313	0.409	8.9 × 10^−8^	0.427	2.9 × 10^−8^	0.412	5.4 × 10^−10^	0.358	4.8 × 10^−10^
318	0.395	1.1 × 10^−7^	0.414	3.6 × 10^−8^	0.357	1.2 × 10^−9^	0.292	1.2 × 10^−9^
323	0.380	1.5 × 10^−7^	0.402	4.4 × 10^−8^	0.298	2.7 × 10^−9^	0.236	2.9 × 10^−9^
328	0.375	1.7 × 10^−7^	0.393	5.1 × 10^−8^	0.240	6.5 × 10^−9^	0.195	5.6 × 10^−9^
333	0.358	2.2 × 10^−7^	0.391	5.4 × 10^−8^	0.200	1.3 × 10^−8^	0.154	1.1 × 10^−8^

**Fig. 3:**
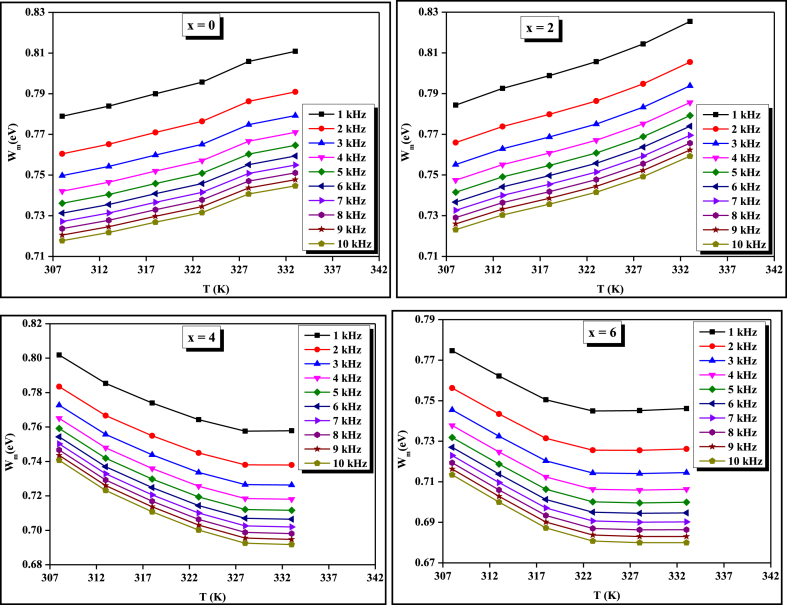
Variation of barrier height with the temperature at different frequencies for STSG glassy alloys.

The study of thermally activated A.C. conduction in the materials provides useful information about the transport phenomena and thoroughly clarifies the convenient model accounting for the conduction mechanism in the chosen samples [28,29]. Literature survey shows that in chalcogenide glasses, the A.C. conduction mechanics is the sum of two types of conduction mechanics namely single polaron and bi-polaron. The experimental data of A.C. conductivity have been fitted to the CBH Model and the values of *NN*_*p*_ are used to fit the theoretically plotted graph of *lnσ*_*ac*_ against 1/*T* withholding to the experimental data. Thus, using the CBH model, the theoretical value of *σ*_*ac*_ was calculated by approaching both the single and bi-polaron hopping. The theoretical and experimental results of *σ*_*ac*_ show that the bi-polaron hopping is dominated over single polaron hopping in our case because the theoretically calculated value of A.C. conductivity followed by the bi-polaron hopping approach lies near to the experimental value in comparison to the single polaron hopping approach. The theoretical basis of these two approaches was reported in the literature [30]. From equation (3), we have calculated the value of *W*_*m*_ using the value of the frequency exponent. The temperature dependence of maximum barrier height (*W*_*m*_) was depicted in Fig. 3. From this figure, it is clear that the value of *W*_*m*_ increases with increasing temperature for ternary x = 0 and quaternary x = 2 compositions however, for a higher concentration of Ge i.e., x = 4 and x = 6, it decreases with temperature.

**Fig. 4:**
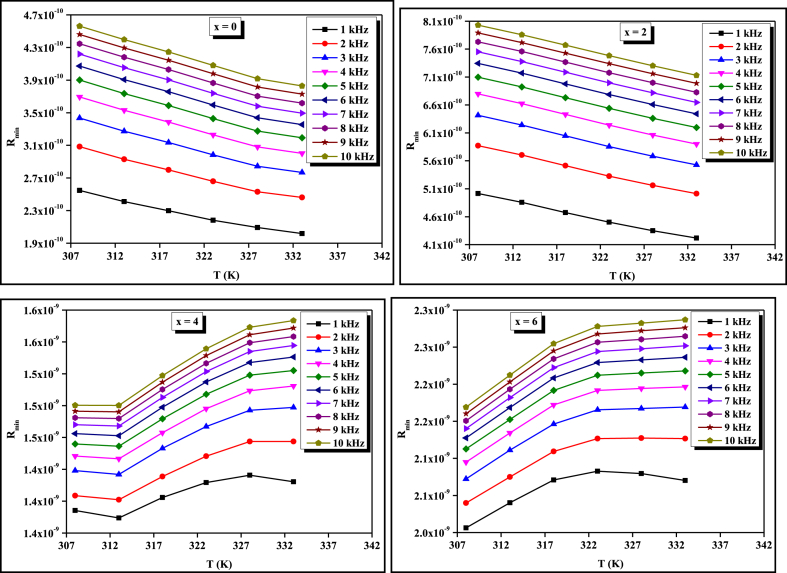
Temperature dependence of minimum hopping distance of bi-polaron for STSG glassy alloys.

**Fig. 5:**
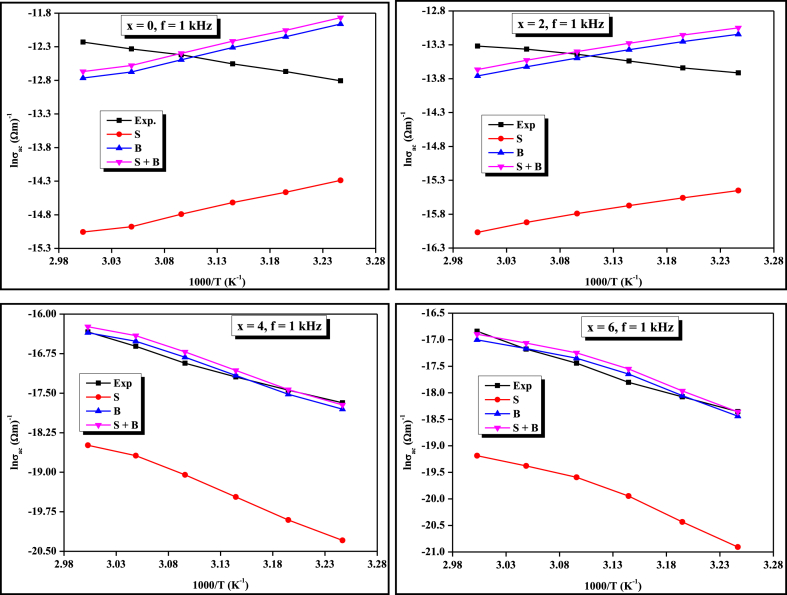
Comparative temperature-dependent plots of theoretical (for single-polaron and bi-polaron) and experimental values of *σ*_*ac*_ at 1 kHz frequency for STSG glassy alloys.

**Fig. 6:**
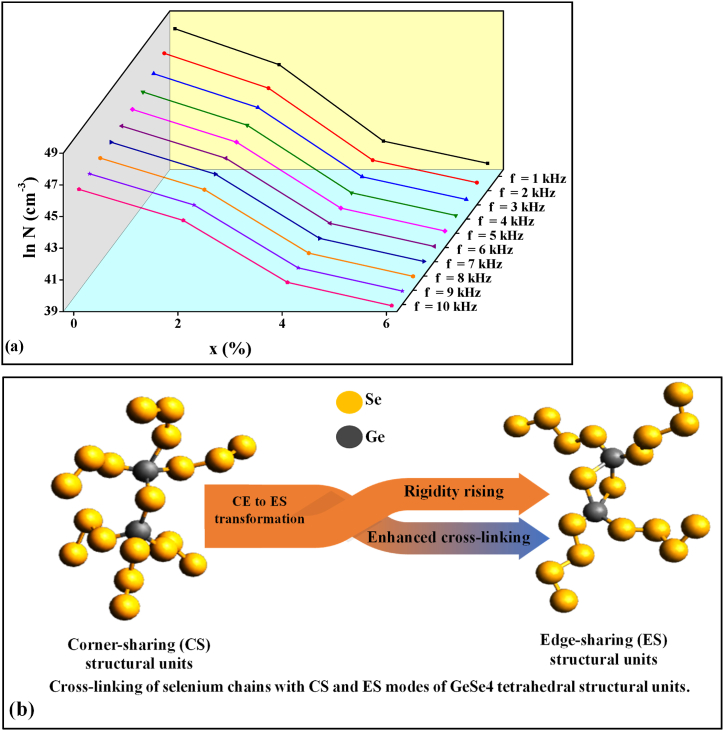
(a) Compositional dependence of DDS at different frequencies, and (b) the possible mechanism behind the significant rise in DDS beyond x = 2.

The cut-off or minimum hopping distance (*R*_min_) can be calculated using equation (4). Its variation with temperature for four compositions is shown in Fig. 4 and values are tabulated in Table 3. Using equations (5) and (7), one can calculate the values of Coulombic barrier height *W* and *R*_*ω*_ respectively. Fig. 5 demonstrates the *σ*_*ac*_ plots against 1/*T* for four compositions at a particular frequency of 1 kHz. Similar graphs are also plotted for other frequencies (i.e., 2 kHz–10 kHz) but are not shown here because the nature of the graphs is the same except some shifting. This figure indicates that there is a good agreement between the calculated value of *σ*_*ac*_ for bi-polaron hopping and experimentally determined values of *σ*_*ac*_ in comparison to single polaron hopping. Murti et al. also found that bi-polaron is dominated over single polaron in the Ge_x_Se_100-x_ system above room temperature [21]. Next, we calculated the value of *N* (i.e., DDS) for bi-polaron hopping and tabulated it in Table 2. This table shows that the DDS decreases insignificantly for composition x = 2. Beyond x = 2, it is reduced noticeably by further increasing the Ge concentration (i.e., x = 4, 6). The compositional variation of DDS at different frequencies is shown in Fig. 6(a). One can see from this figure that the DDS also reduces by enhancing the value of frequencies. The decrement in the value of defect states with Ge concentration can be attributed to the fact that more cross-linking of Se chains takes place in corner-sharing GeSe_4_ tetrahedral units as compared to edge-sharing ones as shown in Fig. 6(b). Since when Ge atoms enter the glass network of the ternary STS system at cost of Se, they form the GeSe_4_ tetrahedral structural unit with Se. For lower concentrations of Ge i.e., (x = 2), the glassy network becomes floppy so its thermal stability, as well as characteristic temperature (*T*_*g*_ and *T*_*c*_), decreases [7] while for higher concentrations of Ge i.e., (x = 4), and (x = 6), the structure becomes more rigid as compared to earlier one i.e., (x = 0) and (x = 2). The pieces of evidence of this fact are observed in different properties of the present glassy series [[6], [7], [8], [9]]. Thus, the DDS is higher at lower concentrations of Ge because at low concentrations, the number density of corner-sharing tetrahedral units is more so more cross-linking of Se chains takes place with them. However, the structural rigidity is increased at higher concentrations of Ge (x = 4, 6) since the number density of edge-sharing tetrahedral units is increased. Thus, the number of long polymeric chains of selenium (… – Se– Se–Se–Se– …) is reduced from six to four. Consequently, the cross-linking of Se chains is improved in the edge-sharing configuration which results in the reduction of the density of defect states at higher concentrations of Ge i.e., (x = 4) and (x = 6). Thus, the cross-linking of corner/edge sharing GeSe_4_ tetrahedral structural units facilitates a way to control the DDS in the present glassy system.

**Table 2:** Values of *R*_*ω*_, and DDS (*N*) for bi-polaron hopping conduction at different frequencies of STSG system at 318 K.

*f* (kHz)	x = 0		x = 2		x = 4		x = 6	
	*R*_*ω*_ (nm)	*N* (cm^−3^)	*R*_*ω*_ (nm)	*N* (cm^−3^)	*R*_*ω*_ (nm)	*N* (cm^−3^)	*R*_*ω*_ (nm)	*N* (cm^−3^)
1	0.7	9.7 × 10^20^	1.3	1.0 × 10^20^	4.4	8.1 × 10^17^	6.9	2.0 × 10^17^
2	0.8	5.0 × 10^20^	1.5	5.6 × 10^19^	4.4	6.0 × 10^17^	6.9	1.4 × 10^17^
3	0.9	3.4 × 10^20^	1.6	4.0 × 10^19^	4.4	5.1 × 10^17^	6.9	1.2 × 10^17^
4	0.9	2.6 × 10^20^	1.7	3.2 × 10^19^	4.4	4.6 × 10^17^	6.9	1.1 × 10^17^
5	1.0	2.2 × 10^20^	1.8	2.8 × 10^19^	4.4	4.2 × 10^17^	6.9	1.0 × 10^17^
6	1.0	1.9 × 10^20^	1.8	2.4 × 10^19^	4.4	4.0 × 10^17^	6.9	9.3 × 10^16^
7	1.0	1.6 × 10^20^	1.9	2.2 × 10^19^	4.4	3.8 × 10^17^	6.9	8.9 × 10^16^
8	1.1	1.5 × 10^20^	1.9	2.0 × 10^19^	4.4	3.7 × 10^17^	6.9	8.5 × 10^16^
9	1.1	1.3 × 10^20^	2.0	1.9 × 10^19^	4.4	3.6 × 10^17^	6.9	8.3 × 10^16^
10	1.1	1.2 × 10^20^	2.0	1.8 × 10^19^	4.4	3.5 × 10^17^	6.9	8.1 × 10^16^

**Table 3:** Values of *R*_min_, for bi-polaron hopping conduction at different frequencies of STSG system at 318 K.

*f* (kHz)	*R*_min_ (nm)			
	x = 0	x = 2	x = 4	x = 6
1	0.2	0.5	1.4	2.1
2	0.3	0.6	1.4	2.2
3	0.3	0.6	1.5	2.2
4	0.3	0.6	1.5	2.2
5	0.4	0.7	1.5	2.2
6	0.4	0.7	1.5	2.3
7	0.4	0.7	1.5	2.3
8	0.4	0.7	1.5	2.3
9	0.4	0.8	1.5	2.3
10	0.4	0.8	1.5	2.3

## 5 Conclusions

We have determined the DDS in STSG glassy system through AC conductivity measurements. The temperature-governed A.C. conduction is governed by correlated barrier hopping. The detailed analysis discloses that bi-polaron hopping contributes the maximum contribution to *σ*_*ac*_, while the contribution of single-polaron hopping is insignificant. The DDS decreases gradually with the rise in frequency but noticeably with increasing concentration of Ge. The rise in the structural rigidity and concentration of edge-sharing GeSe_4_ tetrahedral structural units strengthens the cross-linkage of Se and Ge. Thus, the DDS is decreased with increasing Ge content as a consequence of modification in the concentration of corner/edge sharing GeSe_4_ tetrahedral structural units. This work might offer an innovative approach for preparing different glasses in which DDS can be varied by controlling the cross-linking of foreign Ge atoms in selenium-rich glasses.

## Data availability statement

Data will be made available on request.

## CRediT authorship contribution statement

**S.K. Pal:** Data curation, Formal analysis, Writing – original draft. **A. Dahshan:** Funding acquisition, Resources, Investigation, Methodology. **Neeraj Mehta:** Conceptualization, Investigation, Supervision, Writing – review & editing.

## Declaration of Competing interest

We confirm that the manuscript entitled “Correlation between the density of defect states (DDS) and cross-linking of corner/edge sharing GeSe4 tetrahedral structural units” has no Conflict of Interest.
